# An Immunopharmacoinformatics Approach in Development of Vaccine and Drug Candidates for West Nile Virus

**DOI:** 10.3389/fchem.2018.00246

**Published:** 2018-07-06

**Authors:** Mohammad Uzzal Hossain, Chaman Ara Keya, Keshob Chandra Das, Abu Hashem, Taimur Md. Omar, Md. Arif Khan, S. M. Rakib-Uz-Zaman, Md. Salimullah

**Affiliations:** ^1^Bioinformatics Division, National Institute of Biotechnology, Dhaka, Bangladesh; ^2^Department of Biochemistry and Microbiology, North South University, Dhaka, Bangladesh; ^3^Molecular Biotechnology Division, National Institute of Biotechnology, Dhaka, Bangladesh; ^4^Microbial Biotechnology Division, National Institute of Biotechnology, Dhaka, Bangladesh; ^5^Department of Biotechnology and Genetic Engineering, Life Science Faculty, Mawlana Bhashani Science and Technology University, Tangail, Bangladesh; ^6^Bio-Bio-1 Research Foundation, Sangskriti Bikash Kendra Bhavan, Dhaka, Bangladesh; ^7^Department of Mathematics and Natural Sciences, Biotechnology Program, BRAC University, Dhaka, Bangladesh

**Keywords:** West Nile Virus, immunoinformatics, pharmacoinformatics, vaccine design, drug design

## Abstract

An outbreak of West Nile Virus (WNV) like the recent Ebola can be more epidemic and fatal to public health throughout the world. WNV possesses utmost threat as no vaccine or drug is currently available for its treatment except mosquito control. The current study applied the combined approach of immunoinformatics and pharmacoinformatics to design potential epitope-based vaccines and drug candidates against WNV. By analyzing the whole proteome of 2994 proteins, the WNV envelope glycoprotein was selected as a therapeutic target based on its highest antigenicity. After proper assessment “KSFLVHREW” and “ITPSAPSYT” were found to be the most potential T and B-cell epitopes, respectively. Besides, we have designed and validated four novel drugs from a known WNV inhibitor, AP30451 by adopting computational approaches. Toxicity assessment and drug score confirmed the effectiveness of these drug candidates. This *in silico* research might greatly facilitate the wet lab experiments to develop vaccine and drug against WNV.

## Introduction

West Nile Virus (WNV) belongs to Flaviviridae family which shows the close association with dengue virus. WNV is transmitted by mosquito and causes febrile illness upon infection that further develops encephalitis including flaccid paralysis and cognitive dysfunction (Ceausu et al., 1997; Petersen and Marfin, 2002; Sejvar et al., 2003). Birds are the main target of WNV encephalitis but other vertebrates including humans are also at the edge of high risk. WNV fever and encephalitis have become prevalent throughout the world including the Middle East, Europe, Africa and America since the mid-1990s (Hubalek and Halouzka, 1999; Lanciotti et al., 1999; Petersen et al., 2003; Dauphin et al., 2004; Hayes et al., 2005; Mattar et al., 2005; Deardorff et al., 2006; Komar and Clark, 2006). The symptoms of WNV are more prevalent in elderly and immuno-compromised person although patients might be affected in any stages of age. The survey reported that fatality rate of most recent WNV outbreak is about 4–14% but it might be increased from 10 to 19% in hospitalized cases (Mattar et al., 2005). However, despite WNV's worldwide distribution and epidemic nature, currently, no effective medication is available that can prevent or cure this infection. Therefore, the patients with WNV infection were likely to have supportive treatment such as mosquito net usage, intensive nursing care, painkiller administration etc. (Hayes and Gubler, 2006; Division of Vector-Borne Infectious Disease, 2007).

The enveloped virion (50 nm) of WNV is comprised of RNA genome surrounded by a lipid bilayer membrane. The secondary structure of envelope protein (E) plays a vital role in translation, RNA synthesis, and packaging (Shi et al., 1996; Khromykh et al., 2001; Granwehr et al., 2004; Busch et al., 2005; Friebe and Harris, 2019). The envelope glycoproteins (E) intercede viral attachment and their entry by membrane fusion (Kanai et al., 2006). The domain of E protein is important for eliciting neutralizing antibodies as it is exposed on the viral surface. The envelope E glycoprotein is the foremost antigen which is also the essential target for vaccine development (Roehrig, 2003). The envelope (E) protein selected for drug design plays a crucial role in translation, RNA synthesis and packaging. We have selected a potential inhibitor AP30451 (experimentally validated from 80000 chemical compounds) which inhibits the translation and replication of WNV. The compound AP30451 inhibited translation of WNV mRNA and blocked WNV replication upon inhibitor screening of WNV replicon and Enzyme-linked Immunosorbent Assay (ELISA) of several cell types (Noueiry et al., 2007). And we have designed some inhibitor compounds from parent molecules AP30451. As E protein is essential for translation, it is, therefore, to be selected an inhibitor AP30451 which will ultimately inhibit the translation mechanism of WNV. The structural (capsid, envelope, and pre-membrane) and nonstructural (NS1, NS2A, NS2B, NS3, NS4A, NS4B, and NS5) proteins were found during translation of viral RNA (Lindenbach et al., 2007). Receptor interaction, membrane fusion, and virion assembly are regulated by the envelope (E) protein. The conformation of E protein during virion assembly and premature fusion of it during virus exocytosis to the cell surface are both stabilized by the pre-membrane (prM) (Westaway et al., 2002). prM is cleaved to M (infectious virions that are released by exocytosis) via a furin mediated pathway upon the transportation through the trans-Golgi network (Guirakhoo et al., 1991; Elshuber et al., 2003).

The approach of vaccinomics which is integrated with bioinformatics tools has been better choice for the recent development of new vaccines (Flower, 2008; Poland et al., 2009). To emphasize this, here we propose an integrated approach, relying on vaccine development and drug prediction against WNV. Vaccinomics already demonstrated its potentials against some life-threatening diseases such as multiple sclerosis (Bourdette et al., 2005) malaria (López et al., 2001) and tumors (Knutson et al., 2001) through the development of the vaccine. An ideal vaccine that prompts immunity should essentially be able to eliminate the risk of re-infection as well as to elicit a specific immunological response (Atanas and Irini, 2013). In this perspective, immune dominant B-Cell and T-cell epitopes mapping is the prime concern of immunoinformatics based research to reduce expenses and valuable time for the development of vaccines. The chronic symptoms which may last for months to years might require being treated with the vaccine and drug simultaneously. Moreover, a mutation could shrink the effectiveness of the vaccine/drug candidates against the viral disease. Therefore, concurrent development of vaccine and drug candidates against viral disease might be the interest of the researchers and clinicians. To emphasize this, we have added both vaccine and drug therapy approach in the same study. Still, vaccination might be ineffective due to the emergence of a sudden outbreak of WNV. Post-therapeutics or drugs could be the best option until the new vaccine is developed. Here, we employ an integrated approach relying on both vaccine and drug development against WNV. Computer -aided screening techniques and docking simulation could significantly contribute to determine suitable inhibitors to the target proteins and to find out exclusive binding sites of potential target proteins respectively. Inhibitor molecules for dengue virus, envelope protein have recently been successfully identified based on this assumption methodology (Yennamalli et al., 2009). The approach of bioinformatics in the field of vaccine and drug discovery has already prolifically performed against some virus and bacteria (Sharmin and Islam, 2014; Hasan et al., 2015; Khan et al., 2015; Oany et al., 2015; Hossain et al., 2016a,b,c). Therefore, we have analyzed WNV entire proteome and identified its potential immunogenic regions. Further, computational methods have been performed for the prediction of inhibitor molecules against the active site of the target protein. This *in silico* research has been steered to design effective peptide vaccine and some novel drugs for the better medication system that could lead the possible treatment as well as prevention of WNV infection.

## Materials and methods

### Retrieving west nile virus protein sequences

All the protein sequences of human West Nile virus available in UniProt Knowledge Base (UniProtKB) database (http://www.uniprot.org) (Apweiler et al., 2004; The UniProt Consortium, 2014) were retrieved and then stored in FASTA format for the analysis of immunogenic properties.

### Identification of most potent antigenic protein and its divergence analysis

To identify the most potent antigenic protein, the antigenic value of each protein was assigned using an online prediction server, Vaxijen v2.0 (Doytchinova and Flower, 2007). Protein having highest antigenic value was considered as the most potent antigenic protein. Default parameter of this server was used for this identification.

### Structure analysis of highest antigenic protein

MODELLER 9v11 (Šali et al., 1995) through HHpred (Söding, 2005; Söding et al., 2005) was used to predict the 3D structure of envelope glycoprotein of WNV. The model assessment tools Anolea (Melo et al., 1997), ProCheck (Laskowski et al., 1993), and Verify3D (Eisenberg et al., 1997) were employed to ensure the model quality.

### Vaccine design

#### Prediction of T cell epitope

NetCTL 1.2 (http://www.cbs.dtu.dk/services/NetCTL/) server was utilized for enlisting the interacted Cytotoxic T-lymphocyte (CTL) epitopes. The candidate epitopes were predicted based on the MHC class I supertypes (A1, A2, A3, A24, A26, B7, B8, B27, B39, B44, B58, and B62) and peptide binding (Larsen et al., 2005). The threshold value was set to 0.5 by which we could assess our findings more decisively to generate more epitopes. The best candidates for vaccine development were chosen for further analysis based on a combined score of class I binding, transporter of antigenic peptides (TAP) transport efficiency and proteasomal cleavage prediction.

Stabilized Matrix Method (SMM) (Peters and Sette, 2005; Tenzer et al., 2005) was implemented for the prediction of the suitable epitope from MHC II molecules.

#### Analyzing epitope conservancy

The predicted epitopes were employed in Immune Epitope Database (IEDB) analysis resource to measure the epitope conservancy level within the all protein sequences of WNV (Bui et al., 2007).

#### Population coverage prediction

We have employed IEDB population coverage tool (Bui et al., 2007) to calculate the population coverage of epitopes.

#### Allergenicity appraisal

The allergenicity of proposed epitopes was assessed by a cross-reactive allergen prediction program, AllerHunter. Prediction of allergen and non-allergen epitopes were determined by AllerHunter with high sensitivity and specificity (Liao and Noble, 2003; Muh et al., 2009).

#### Design of the three-dimensional (3D) epitope structure

We have utilized PEP-FOLD server (Thevenet et al., 2012) to predict the 3D structure of the most prospective epitope represented with a 9-mer peptide sequence “KSFLVHREW” to analyse the binding affinity with Human Leukocyte Antigens (HLAs). The server proposed five 3D structures of corresponding epitope but only one was selected for the interaction with HLAs.

#### Docking simulation study

Docking experiments were performed by Autodock Vina (Trott and Olson, 2010) to reveal out the binding affinity between predicted epitopes and HLA molecules. The HLA-B^*^35:01 (3LKN) molecule was retrieved from Research Collaboratory for Structural Bioinformatics (RCSB) (Berman et al., 2000) and further subjected to Discovery studio (Van Joolingen et al., 2005) to remove the NP418 epitope which was complexed with HLA molecule. In order to compare with the previous binding affinity NP418 epitope and prepared HLA-B^*^35:01 was also performed in docking study. Moreover, 1D5M crystal structure was also retrieved to perform docking study with the selected epitope for Major Histocompatibility Complex II (MHC-II) molecule binding affinity.

#### Identification of the B cell epitope

To initiate an immune response, B cell epitope plays a vital role by interacting with B lymphocytes (Nair et al., 2002). IEDB tools were utilized to confirm the antigenic properties of B-cell epitope such as Kolaskar and Tongaonkar antigenicity scale (Kolaskar and Tongaonkar, 1990), Emini surface accessibility prediction (Emini et al., 1985), Karplus and Schulz flexibility prediction (Karplus and Schulz, 1985), Bepipred linear epitope prediction analysis (Larsen et al., 2006) and Chou and Fasman beta-turn prediction analysis (Chou and Fasman, 1978; Rini et al., 1992).

#### Prediction of epitope hydrophilicity

The selected epitopes were analyzed to evaluate the hydrophobic properties by using the Parker hydrophilicity prediction tool (Parker et al., 1986) of Immune Epitope Database (IEDB) in which default threshold of 3.448 was used.

### Drug development

#### Binding site analysis

The binding site of the constructed model was identified by using the meta-pocket server 2.0 (Bingding, 2009) and Discovery studio (Van Joolingen et al., 2005). The predicted binding site obtained by these servers could be employed for the discovery of effective drugs against envelope glycoprotein.

#### Analyzing active site

To convey noteworthy insight on drug-protein interactions active site analysis was carried out by using Computed Atlas of Surface Topography of proteins (CASTp) server (Dundas et al., 2006). The active binding sites were identified based on the structural similarity between template and model.

#### Selection of control compound and designing of its analogues

National Center for Biotechnology Information, NCBI (http://www.ncbi.nlm.nih.gov), Public Medline, Pubmed (www.ncbi.nlm.nih.gov/pubmed) and Google Scholar (http://scholar.google.com/) were searched to find out the inhibitor those are validated with the lab experiment. Therefore, the AP30451 compound was selected for our study as a control compound. Replication dependent luciferase expression and Adenosine Triphospate (ATP) quantification assay of AP30451 were previously performed and reported to assess its antiviral activity and cytotoxic effect on WNV (Noueiry et al., 2007). For the advancement of drug nature of this known inhibitor molecule, novel drug molecules were generated by employing ACD/Chemsketch (Fan et al., 2002) and Molinspiration (Mishra and Raghava, 2011). Thereafter, 2D structure data files (SDF) were stored as Mol file for further analysis.

#### Preparation of drug molecules

The Mol file of AP30451 and designed molecules were converted to Protein Data Bank (PDB) file by using Open Bable (OL Boyle et al., 2011). Afterward, the 3D structure optimization of these compounds was accomplished by the ACD/Chemsketch (Fan et al., 2002).

#### Energy minimization of projected model and designed analogues

For homology modeling and designed structures, an accurate alignment is an indispensable requirement to abate the impairment of residues during structure prediction. We have calculated the relative binding free energies for these complexes using the Yet Another Scientific Artificial Reality Application (YASARA) server (Sippl, 1993; Vriend and Sander, 1993; Kuszewski et al., 1997; Krieger et al., 2009).

#### Quantitative structure activity relationship (QSAR) studies

Some sets of QSAR prediction tools Click2drug (http://www.click2drug.org/), Zinc database (http://zinc.docking.org/), Osiris property explorer (Sander, 2001), Molinspiration (Mishra and Raghava, 2011), and AcTor (Judson et al., 2008) were scrutinized to explore the structural properties of drugs.

#### Protein-ligand docking

The energy minimized homology model and the designed analogs with control compound were then performed in Autodock 4.2 (Duan et al., 2003; Morris et al., 2009) and Autodock vina (Trott and Olson, 2010) for docking experiments in which water molecules were omitted but added the polar hydrogen in built model. In docking experiment with autodock vina, a pdbqt file was generated by following grid box parameter (size 48 × 48 × 78 points and center with −14.359 × −6.818 × −39.272, and grid spacing 0.5°A as well). Besides, kollman united atom charges, genetic algorithm (LGA) and population size 150 were utilized in autodock 4.2 for docking studies. These docking experiments were preferred to generate the intermolecular energy, internal energy, of ligand and torsional free binding energy between built model and inhibitors. The molecular visualization of protein-ligands were analyzed by PyMol (Daniel and Bert, 2010), Yasara (Krieger et al., 2009), RasMol (Sayle and Milner-White, 1995), and Discovery studio (Van Joolingen et al., 2005).

#### Pharmacoinformatics studies

The Osiris property explorer (Sander, 2001), Molinspiration (Mishra and Raghava, 2011), ACToR (Aggregated Computational Toxicology Resource) (Judson et al., 2008), ACD/I-Lab (https://ilab.acdlabs.com/iLab2/) and admetSAR (absorption, distribution, metabolism, excretion, and toxicity Structure-Activity Relationship database) (Cheng et al., 2012) were utilized for the calculation of ADME (Absorption, Distribution, Metabolism, Excretion) pharmacological properties and toxic profile. These properties confer the pharmacological activity of the compounds as drug candidates as well as their mode of action within the tissues.

## Results

**Figure 1:**
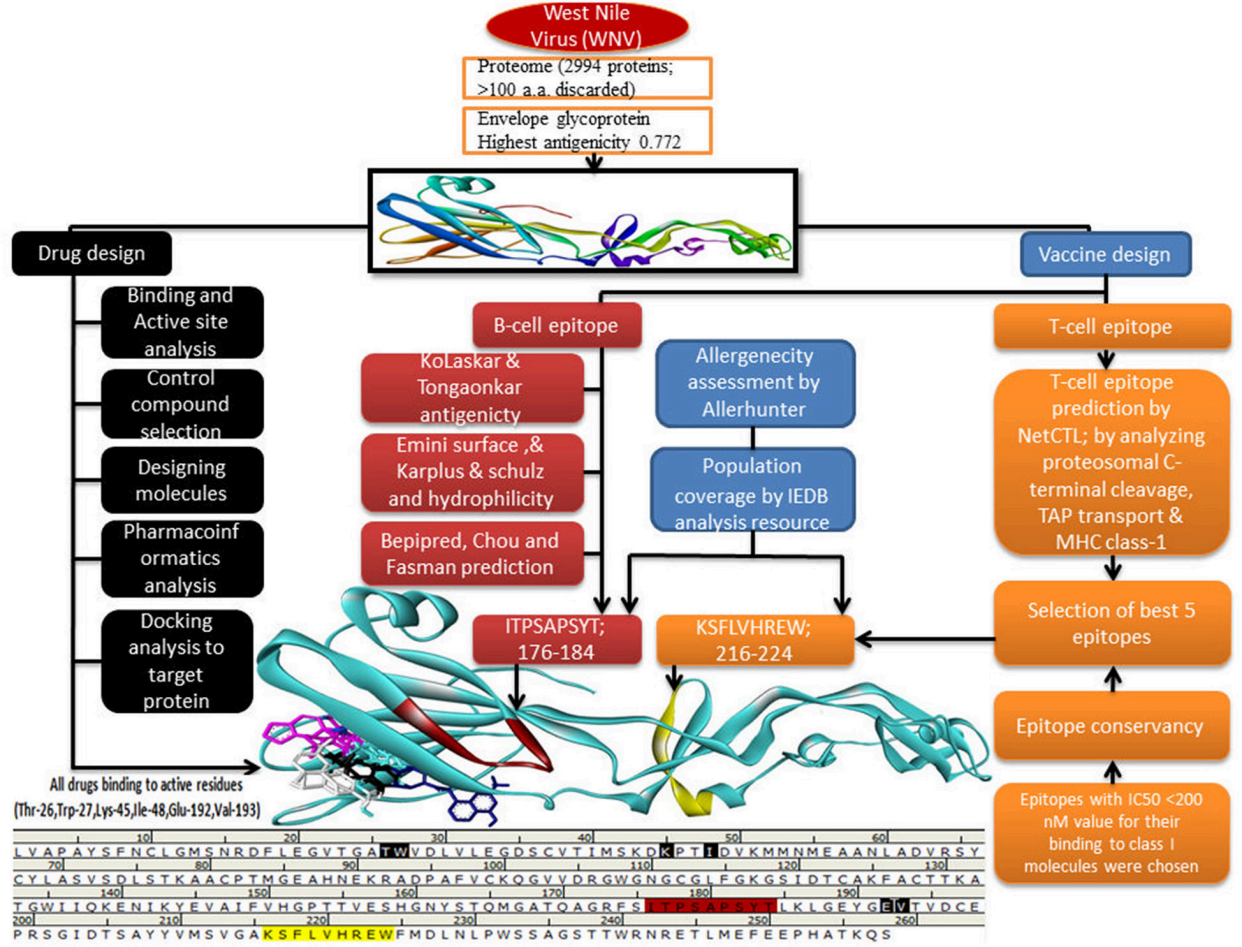
Graphical outline of the current study.

The outline of the current study was shown in Figure 1.

### Vaccine design

#### Antigenicity and divergence analysis of west nile virus proteome

The proteome of WNV composed of 2994 proteins including envelope glycoprotein (758), Genome polyprotein (978), Polyprotein (1345), NS3, NS5 etc. was retrieved as FASTA format and saved in Excel sheet Supplementary Excel File 1. Non-structural proteins and proteins having less than 100 amino acid sequences were excluded from this study. Proteins were most unlikely to prioritize essential proteins when they are less than 100 amino acids (Kumar et al., 2010; Haag et al., 2012). These mini proteins (>100 a.a) have significant role in numerous biological phenomenon and regulatory purposes (Wang et al., 2008). But these mini proteins were deleted from the remaining set of proteins as they are less likely to correspond to the essential therapeutics candidate. In addition to this, the larger amino acid sequence has the possibility to be categorized as promising vaccine/drug targets (Kumar et al., 2010; Haag et al., 2012) Analysis of all the retrieved protein sequences in the VaxiJen server revealed the most antigenic protein as the envelope glycoprotein (UniProtKB id: F1CFF2) with highest prediction score of 0.772 Supplementary Excel File 1.

### Homology modeling and validation

The 3D structure of the highest antigenic envelope glycoprotein was built by employing Modeller 9v11 Supplementary Figure S1. The PSI blast that identified 3P54_A template gives the best coverage of the selected protein. The Ramachandran plot was analyzed to evaluate the quality of structure and it showed 92.9% residues in most favored regions Supplementary Figure S2 and Supplementary Table S1. VERIFY3D and ANOELA also ensured an appropriate 3D structure of Envelope glycoprotein shown in Supplementary Figures S3, S4.

#### T cell epitope identification and conservancy

Five (5) Cytotoxic T-Cell epitopes (CTL) epitopes namely LADVRSYCY, TTVESHGNY, SGIDTSAYY, REWFMDLNL, and KSFLVHREW were selected on the basis of their higher NetCTL combinatorial score Table 1. The MHC-1 molecules were selected followed by half-maximal inhibitory concentration (IC50) value < 200 nm which assures the higher binding capability of selected epitopes to MHC-1 molecules Table 1. The 9-mer epitopes KSFLVHREW and SGIDTSAYY demonstrated the affinity for highest 13 MHC-I molecules Table 1. The conservancy of epitopes from IEDB analysis resource is the essential criteria to induce effective immunogenicity. In our analysis, epitope REWFMDLNL and KSFLVHREW were found with high conservation rate (>82%) whereas epitopes SGIDTSAYY, LADVRSYCY, and TTVESHGNY showed moderate to low conservation rate (5–33%) Table 1. We have also predicted MHC-II molecules of the “KSFLVHREW” as it showed higher conservancy and capability to interact with maximum interacting MHC-I molecules Table 2.

**Table 1:** 5 Potential T-cell epitopes with properties.

**Epitope**	**Interacting MHC-1 allele with an affinity < 200 nm (Total score of proteasome score, Tap score, MHC score, Processing core and MHC-1 biding)**	**Epitope conservancy (%)**
LADVRSYCY	HLA-C^*^12:03 (2.28) HLA-C^*^05:01 (1.56) HLA-A^*^68:23 (1.37) HLA-B^*^35:01 (1.06) HLA-A^*^32:07 (1.04) HLA-B^*^40:13 (0.89) HLA-A^*^32:15 (0.88) HLA-B^*^27:20 (0.76) HLA-C^*^03:03 (0.49) HLA-A^*^01:01 (0.46)	16.67
TTVESHGNY	HLA-A^*^68:23 (1.93) HLA-A^*^26:02 (1.21) HLA-C^*^12:03 (1.09) HLA-B^*^27:20 (1.02) HLA-B^*^15:17 (0.99) HLA-C^*^03:03 (0.80) HLA-B^*^15:02 (0.77) HLA-A^*^32:07 (0.70) HLA-C^*^14:02 (0.58) HLA-A^*^32:15 (0.40) HLA-B^*^40:13 (0.27) HLA-A^*^26:01 (0.26)	33.33
SGIDTSAYY	HLA-A^*^68:23 (1.36) HLA-A^*^32:07 (1.30) HLA-C^*^03:03 (1.28) HLA-C^*^12:03 (1.13) HLA-B^*^40:13 (0.66) HLA-B^*^27:20 (0.61) HLA-B^*^15:02 (0.54) HLA-A^*^30:02 (0.49) HLA-C^*^14:02 (0.42) HLA-B^*^35:01 (0.36) HLA-A^*^29:02 (0.35) HLA-B^*^15:03 (0.28) HLA-B^*^15:01 (0.27)	5.56
REWFMDLNL	HLA-B^*^40:02 (1.03) HLA-B^*^27:20 (0.90) HLA-B^*^40:01 (0.55) HLA-B^*^40:13 (0.49) HLA-A^*^32:07 (0.36) HLA-A^*^02:50 (0.21) HLA-A^*^68:23 (0.12) HLA-C^*^12:03 (−0.12) HLA-A^*^32:15 (−0.23)	82.33
KSFLVHREW	HLA-B^*^15:17 (1.49) HLA-B^*^40:13 (1.44) HLA-B^*^27:20 (1.38) HLA-B^*^58:01 (1.37) HLA-B^*^35:01 (1.31) HLA-C^*^12:03 (0.93) HLA-C^*^15:02 (0.68) HLA-A^*^32:01 (0.66) HLA-A^*^02:50 (0.49) HLA-A^*^68:23 (0.40) HLA-A^*^32:15 (0.36) HLA-A^*^32:07 (0.33) HLA-C^*^07:01 (−0.24)	82.46

**Table 2:** MHC–II molecules from the selected peptide epitope.

**Epitope**	**Interacting MHC-II allele with an affinity < IC50 200 nm**
KSFLVHREW	HLA-DPA1^*^01:03/DPB1^*^02:01(0.04) HLA-DPA1^*^01/DPB1^*^04:01(0.31) HLA-DRB1^*^07:01(1.70) HLA-DQA1^*^05:01/DQB1^*^03:01(20.84) HLA-DRB1^*^01:01(23.91)

#### Prediction of population coverage

**Figure 2:**
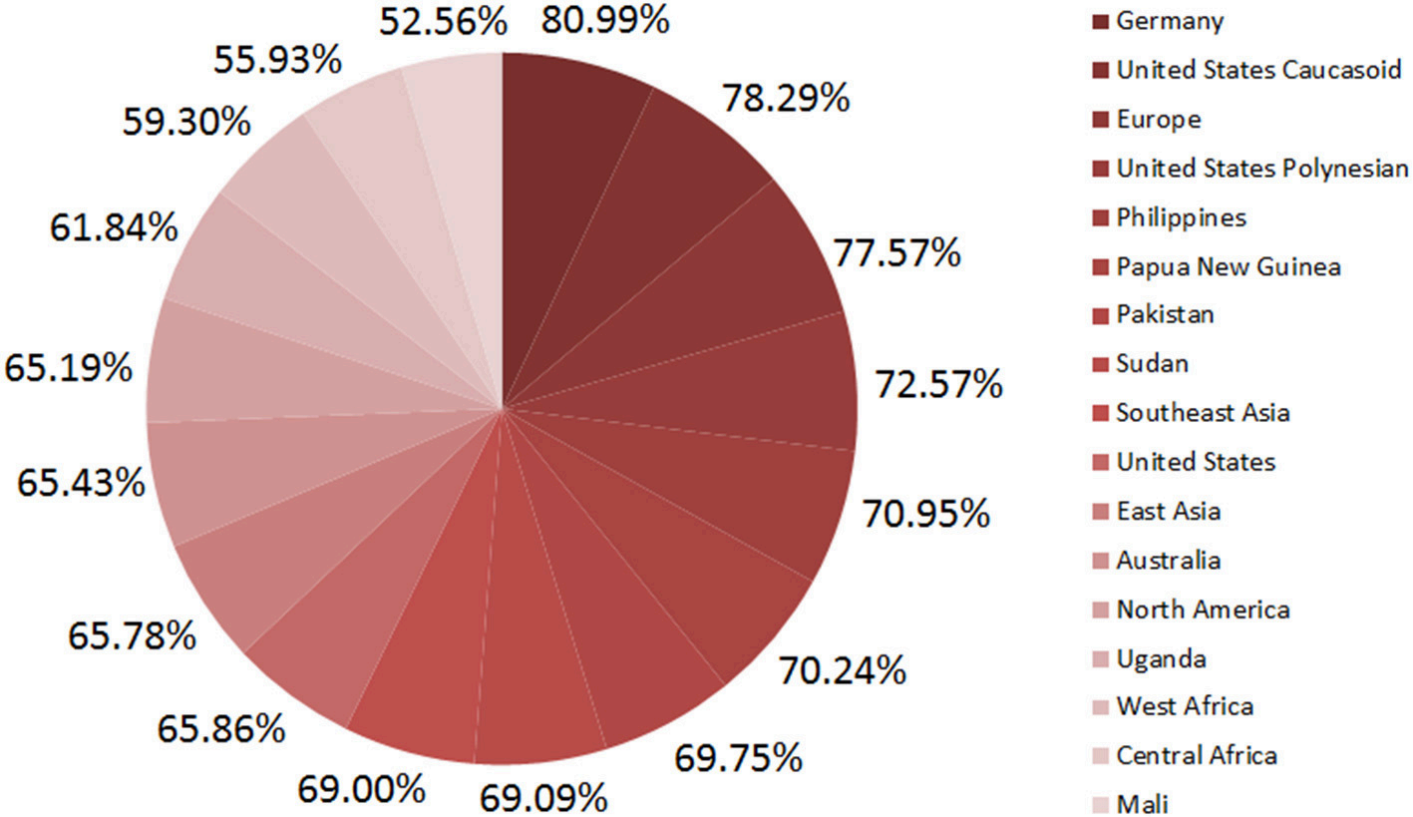
Population coverage of predicted epitopes.

Population coverage measures how many MHC-I molecules prone to respond to predicted epitopes among the people (in percentage) living in a given area. The highest population coverage 81% of epitopes from IEDB was observed in Germany whereas Mali showed the lowest (52.6%) Figure 2. Therefore, at least more than half of the world's population could be covered by these epitopes.

#### Allergenicity assessment

AllerHunter was utilized to predict the sequence-based allergenicity of the epitopes. The analysis with 91.6% sensitivity and 89.3% specificity revealed non-allergenicity of the query epitopes.

#### Molecular docking analysis

**Figure 3:**
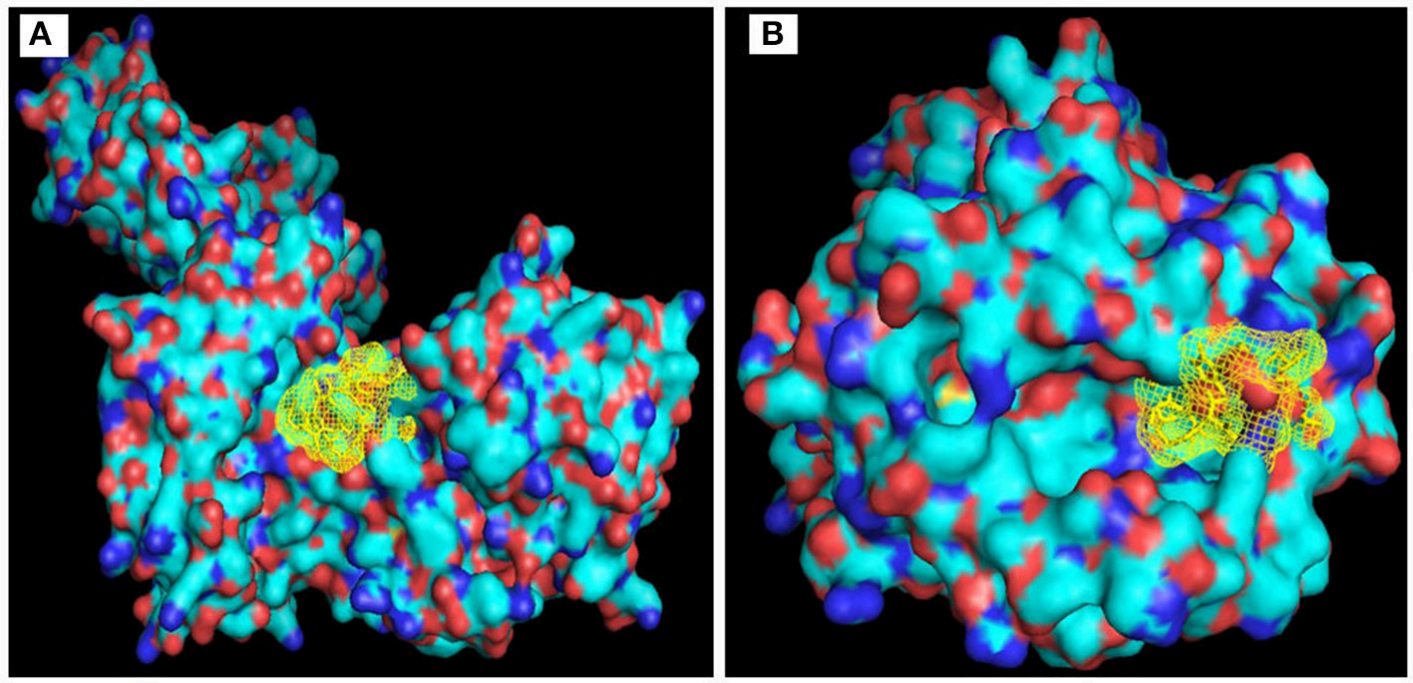
Visualization of binding of KSLVHREW epitope with **(A)** MHC-I (HLA B*35:01); and **(B)** MHC-II (HLA-DR).

AutoDock Vina generated binding mode between “KSFLVHREW” and selected HLA molecules. The binding energy between selected epitope and HLA-B^*^35:01 showed −7.5 kcal/mol which appeared very much similar to influenza NP418 epitope's binding energy −7.6 kcal/mol Figure 3A. The binding energy −7.1 kcal/mol of HLA-DR with the desired epitope was also promising Figure 3B.

#### B cell epitope identification

**Figure 4:**
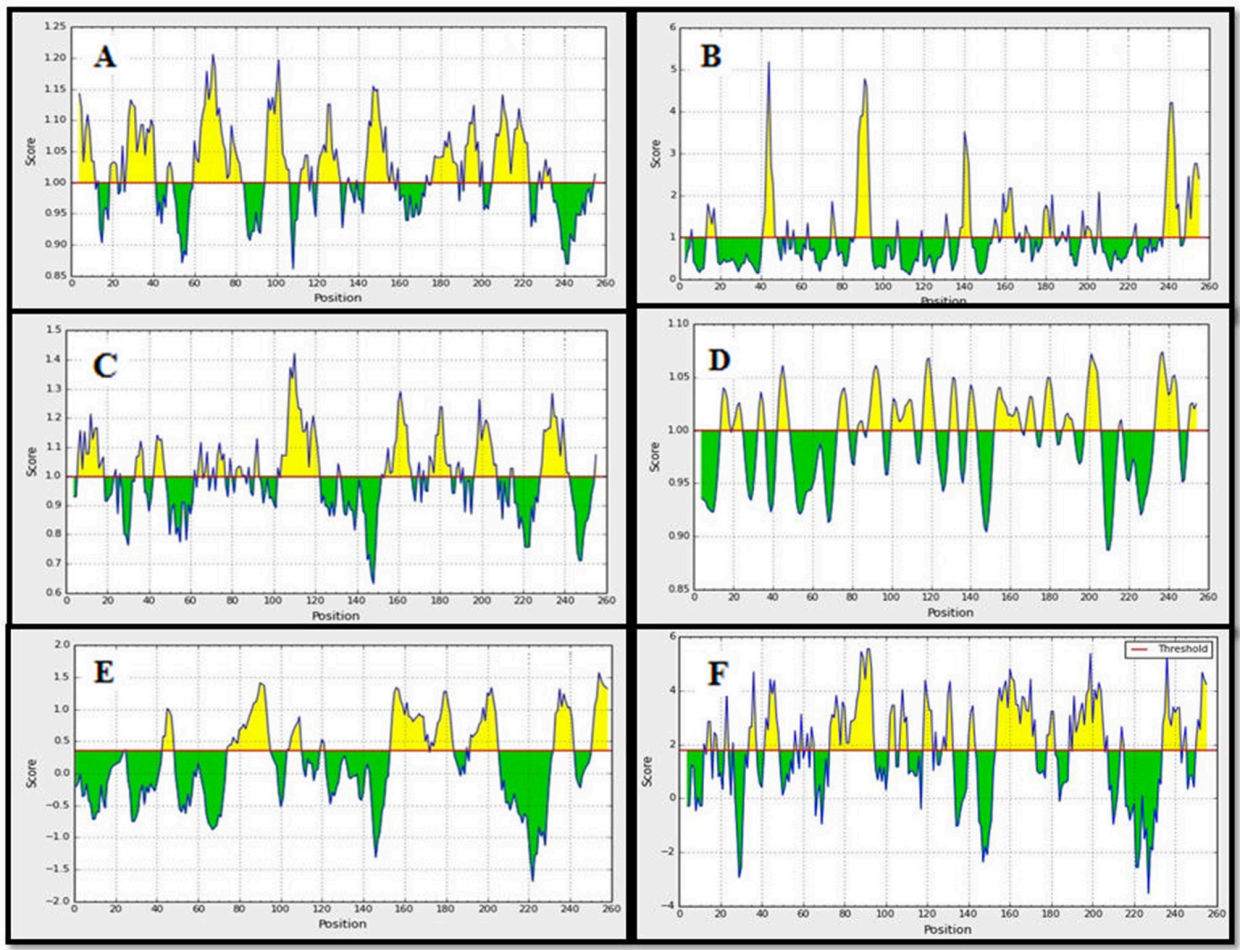
Prediction of B-cell antigenic properties for most antigenic conserved region. Epitope ITPSAPSYT (176–184) showed all the antigenic criteria to be predicted as B-cell epitope. **(A)** Kolaskar and Tongaonkar antigenicity prediction. **(B)** Emini surface accessibility prediction. **(C)** Chou and Fasman beta-turn prediction. **(D)** Karplus and Schulz flexibility prediction. **(E)** Bepipred linear epitope prediction. **(F)** Parker hydrophilicity prediction. The x-axis and y-axis represent the sequence position and corresponding antigenic properties score, respectively. The threshold level is 1.0 for most of the properties except for **(E)** (0.40) and **(F)** (0.1772). The regions having antigenic properties are shown in yellow color above the threshold value.

**Figure 5:**
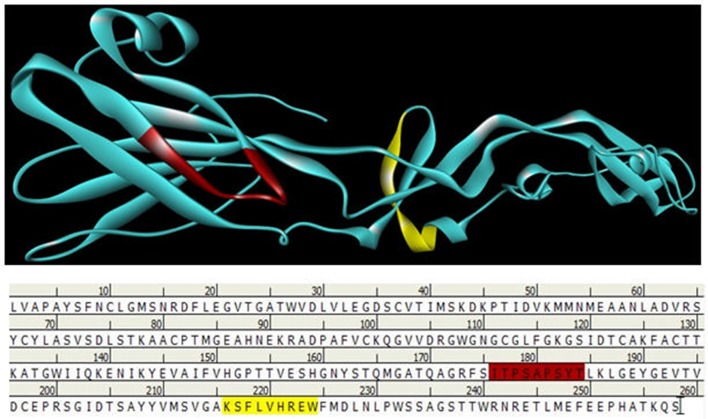
B and T cell epitopes depiction of envelope glycoprotein. Here red color indicates B cell epitope in 176–184 regions and Yellow color indicates T cell epitope is 216–224 regions.

We have implemented some sets of authentic bioinformatics tools to recognize the potential B-cell epitopes. The conserved region were assessed based on physico-chemical properties by Kolaskar and Tongaonkar antigenicity prediction tool. The tool predicted an average antigenic propensity value 1.058 in the scale of maximum value 1.240 and minimum value 0.920. The threshold for antigenicity in the conserved region was 1.00 whereas all values >1.00 from the result considered as potential antigenic determinants. We identified an epitope that meets the threshold criteria with the potentiality to initiate B cell response Figure 4A. Among others, we have found a region of amino acid residues (176–184) as surface accessibility to be urged for potential B cell epitope Figure 4B. The antigenic region of a protein generally contains beta turns which are usually surface accessible and exhibit hydrophilic nature. The region 107–115, 176–184, and 230–238 were reported as the β-turns region from Chou and Fasman Beta-turn prediction Figure 4C. It is speculated that the flexibility of the peptide has a strong correlation with antigenicity (Doytchinova and Flower, 2007). Karplus and Schulz flexibility prediction tool suggested the region of 176–184 as most flexible Figure 4D. Finally, we implemented the Bepipred linear epitope prediction tool to identify the region which could be predicted as the linear B-cell epitope Figures 4E, 5. To confirm the hydrophilic nature of a peptide region, Parker Hydrophilicity was also employed in the region. To analyze the all data from B-cell epitope prediction tools, the conserved sequence “ITPSAPSYT” of 176–184 region was shown to be able to prompt the immunity as B-cell epitope.

### Drug design

#### Binding and active site of envelope glycoprotein

**Figure 6:**
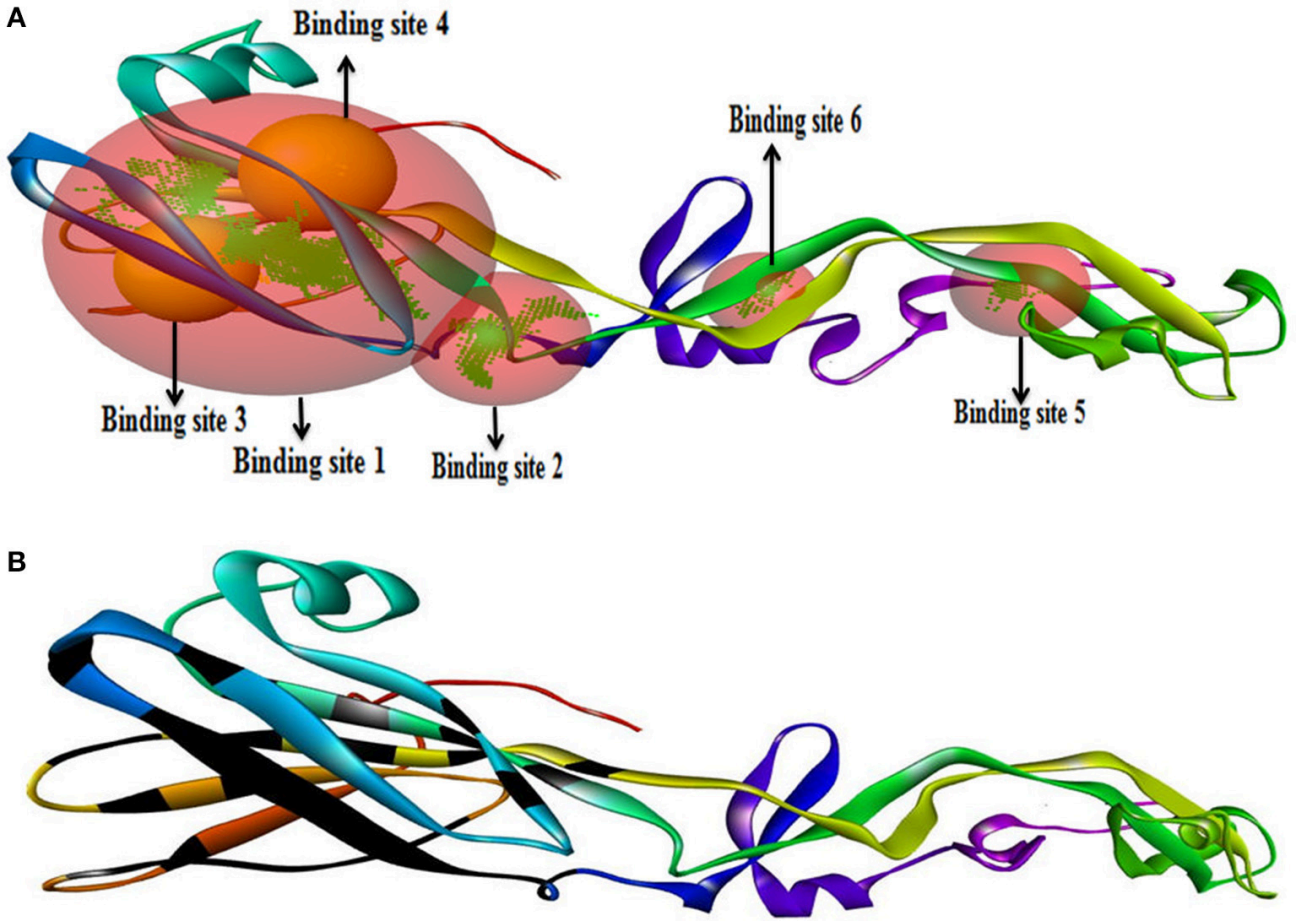
Binding site of envelope glycoprotein **(A)** Active site amino acid residues of envelope glycoprotein. **(B)** The active site residues indicated with black color within envelope glycoprotein of WNV.

We have found six possible binding sites of the modeled 3D protein structure Figure 6A. Then, the CASTp server identified the active site of the envelope glycoprotein. This gives an important prediction about the interacting sites on protein with the ligand molecules Figure 6B.

#### Designing and energy minimization of WNV inhibitors

**Figure 7:**
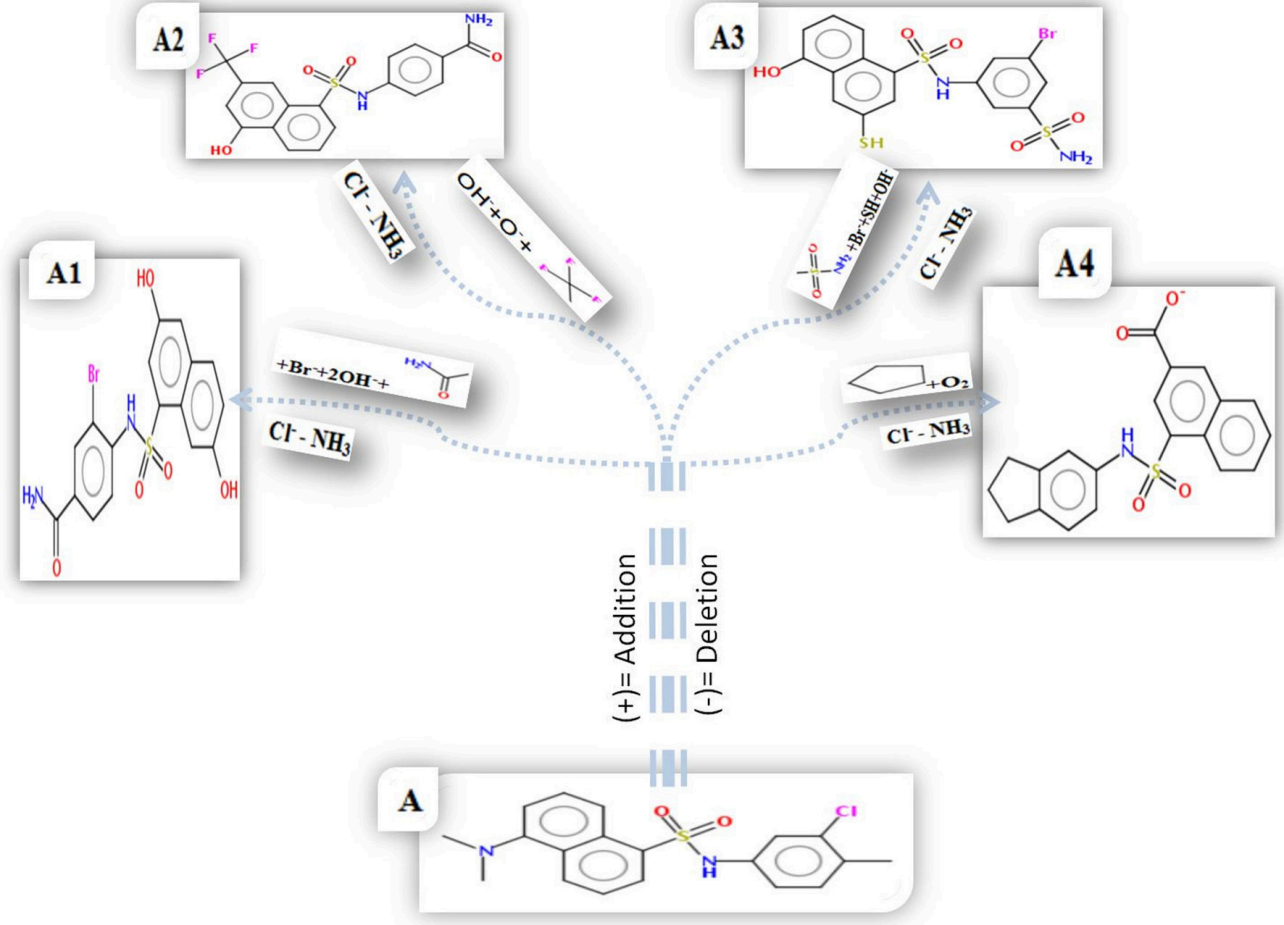
2D structure of parent compound AP30451 and design molecules. **(A)** Parent compound AP30451. Here, Cl^−^ and NH_3_were deleted from the original compounds to generate the novel compounds as WNV inhibitors. **(A1)** Br^−^ and 2OH^−^ were added in designed molecule-1. **(A2)** OH^−^, O^−^ and CF_3_ added in design molecule-2. **(A3)** Br^−^, OH^−^, SH etc. were added in designed molecule-3. **(A4)** O_2_ and a ring structure added in designed molecule-4.

**Figure 8:**
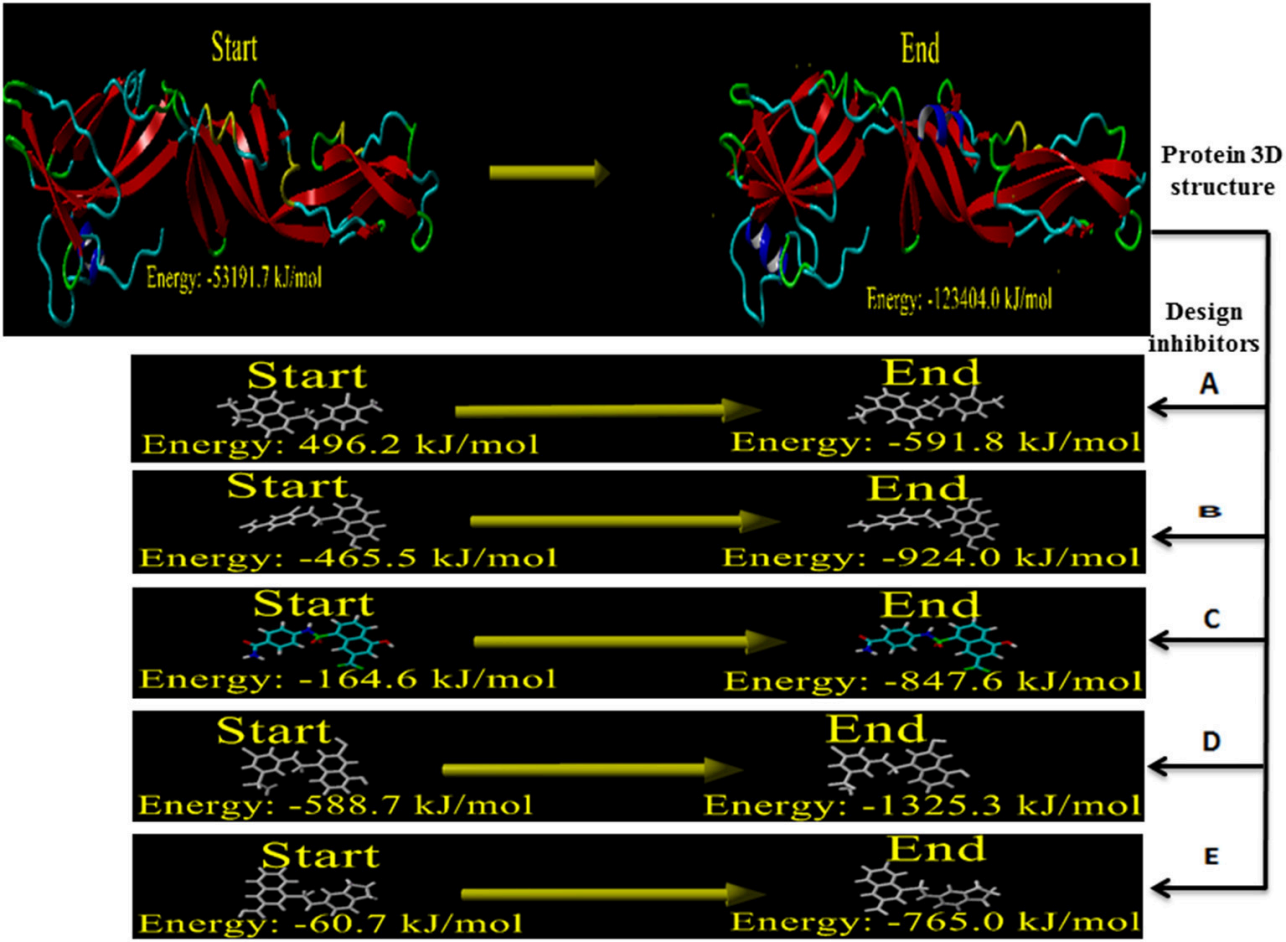
Energy minimization of built model of envelope glycoprotein and designed inhibitors.

The structural defect such as physical realism, stereochemistry, side-chain accurateness, and gap alignment might arise during homology modeling and designing molecules. Therefore, YASARA program was used for the minimization of these structural features which could offer the proper structural stability by yielding the energy (START vs. END) of the constructed model and designed inhibitors Figure 7. The energy comparison between the End energy (−123404.0 kJ/mol) and START energy (−53191.7 kJ/mol) of constructed model confirmed the accurateness of structural features. Besides, the energy comparison of designed molecule 1 (START −465.5 KJ/mol to END −924.0 KJ/mol), designed molecule 2 (START −164.6 KJ/mol to END −847.6 KJ/mol), designed molecule 3 (START −588.7 KJ/mol to END −1325.3 KJ/mol), and designed molecule 4 (START −60.7 KJ/mol to END −765.0 KJ/mol) ensures the structural stability Figure 8.

#### Docking analysis of the designed inhibitors

**Figure 9:**
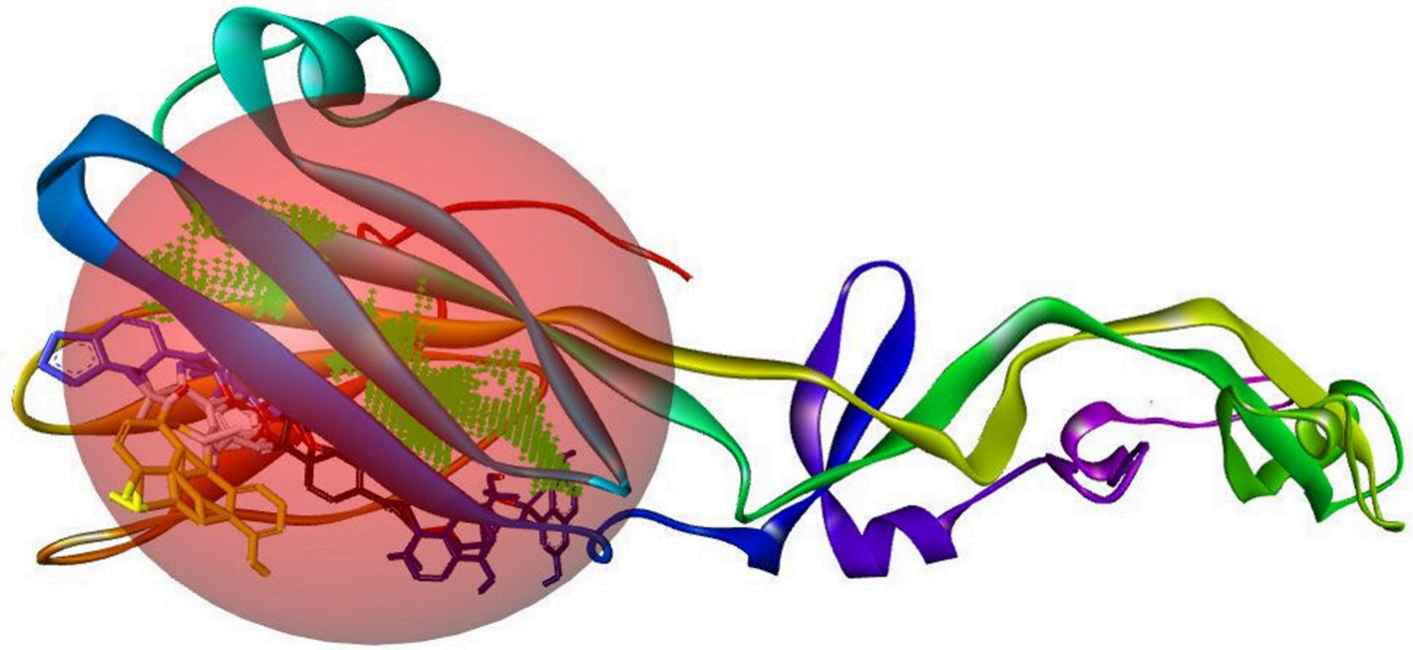
All the designed inhibitors bind in the binding site 1 of the envelopeglycoprotein along with AP30451. The color code of the inhibitors: AP30451 (White); designed molecule 1 (Blue); designed molecule 2 (Yellow); designed molecule 3 (red); designed molecule 4 (Indigo).

**Figure 10:**
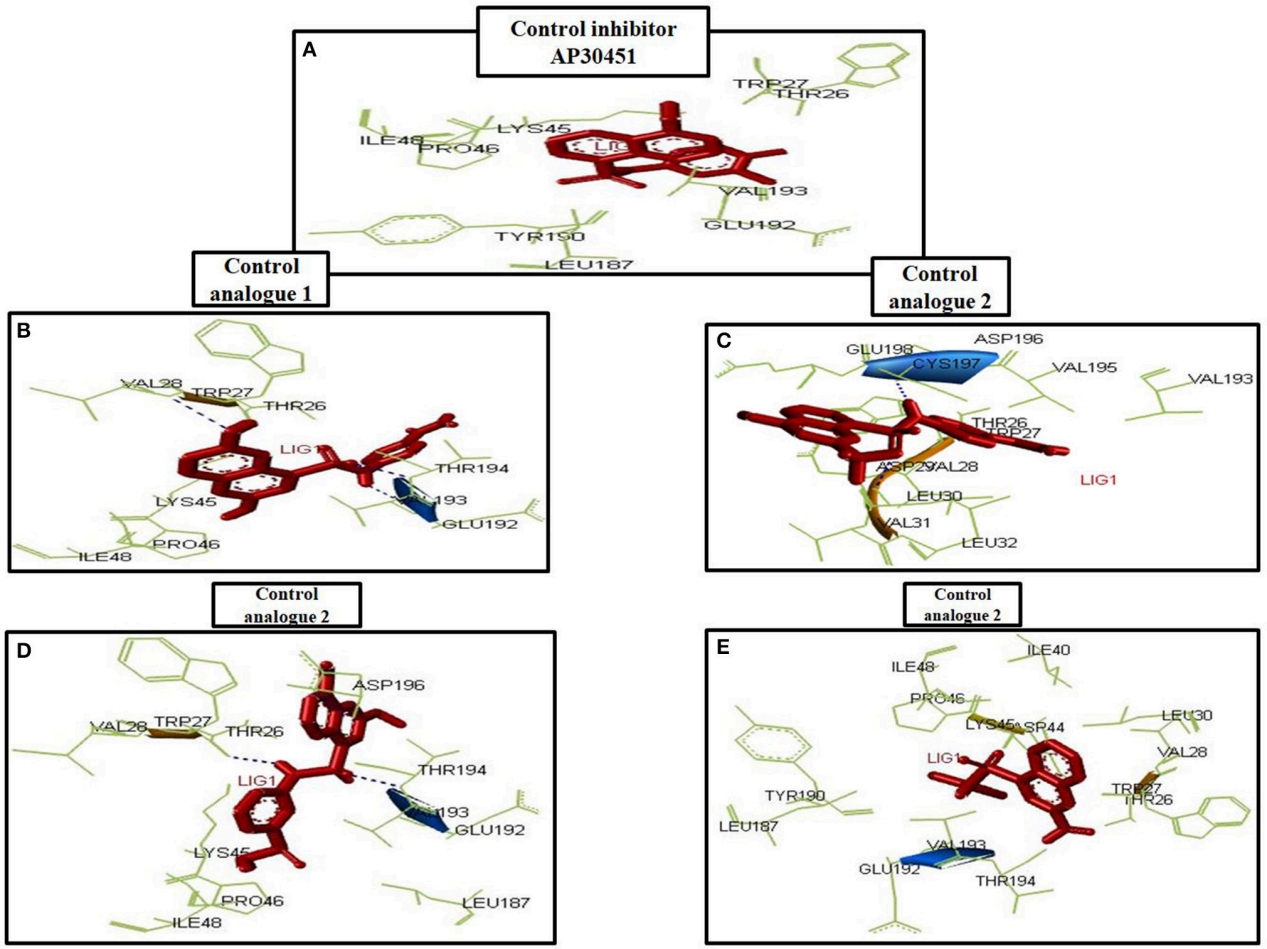
Amino acid interaction with inhibitors. **(A)** AP30451, **(B)** Designed molecule 1, **(C)** design molecule 2, **(D)** Designed molecule 3, **(E)** Designed molecule 4.

**Figure 11:**
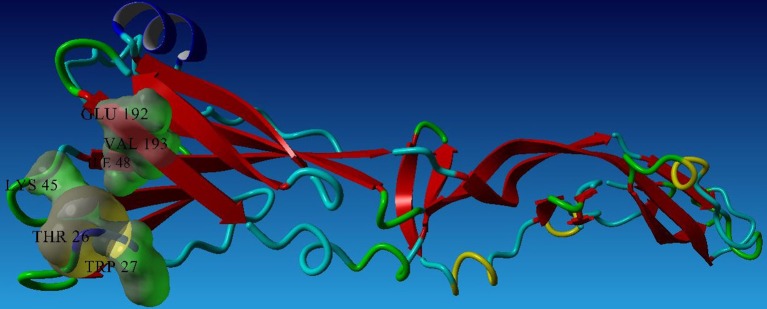
Most common interacting residues of envelope glycoprotein.

To analyze the protein-drug interactions we have performed the molecular docking experiment with two docking software, namely Autodock 4.2 and Autodock Vina of MGL 1.5.6. The docking energy of AutoDock Vina (AP30451: −5.9 KJ/mol, designed molecule 1: −8.0 KJ/mol, designed molecule 2: −8.7 KJ/mol, designed molecule 3: −7.7 KJ/mol and designed molecule 4: −8.9 KJ/mol) and AutoDock 4.2 (AP30451: −4.93 KJ/mol, designed molecule 1: −7.89 KJ/mol, designed molecule 2: −7.67 KJ/mol, designed molecule 3: −6.99 KJ/mol and designed molecule 4: −7.03 KJ/mol) confirmed that they possess strong binding affinity with the target into the binding site Table 3 and Figure 9. Importantly, the interacting amino acid residues from the binding site were also found as active residues Figure 10. The most common interacting amino acid residues were found viz **Thr 26, Trp 27, and Val 193** from control and all the inhibitor molecules Table 4 and Figure 11.

**Table 3:** QSAR properties of control drug and proposed WNV inhibitors.

**Ligand properties**	**AP30451**	**Design molecule 1**	**Design molecule 2**	**Design molecule 3**	**Design molecule 4**
SMILES ID	CN(C)c1cccc2c(cccc12) S(= O)(= O) Nc1ccc(C)c(Cl)c1	NC(= O)c1ccc(NS(= O) (= O)c2cc(O) cc3ccc(O) cc23) c(Br)c1	NC(= O)c1ccc(NS(= O)(= O) c2cccc3c(O)cc(cc23) C(F)(F)F)cc1	NS(= O)(= O)c1cc(Br) cc(NS(= O) (= O)c2cc(S) cc3c(O)cccc23)c1	O = C([O-])c1cc(c2ccccc2c1) S(= O) (= O)Nc1cc c2CCCc2c1
Molecular weight(g/mol)	374.88	437.26	410.37	489.39	367.42
No. of H donor	1	5	4	4	2
No. of H acceptor	4	7	6	7	5
No. of Rotatable Bonds	4	4	5	4	4
cLogP	0.731	0.938	0.905	0.94	0.824
LogS	0.338	0.528	0.468	0.25	0.403
TPSA	57.79	138.1	117.87	182.12	91.85
Drug likeness	−6.39	−0.25	−4.87	−5.82	−2.78
Drug score	0.06	0.45	0.31	0.14	0.31

**Table 4:** Docking result of Designed inhibitors.

**Inhibitors**	**Residues involved**	**Residues name**	**Docking energy/binding affinity (Kcal/mol)**		**Inhibitor constant Ki**	**Internal energy**	**Electrostatic energy**
			**Autodock Vina**	**Autodock 4.2**			
AP30451	9	Thr-26, Trp-27, Lys-45, Pro-46, Ile-48,Leu-187, Tyr-190, Glu-192, Val-193	−5.9	−4.93	37.89	−5.67	−1.89
Designed molecule 1	9	Thr-26, Trp-27, Val-28, Lys-45, Pro-46, Ile-48, Glu-192, Val-193, Thr-194	−8.0	−7.89	46.84	−6.77	−1.80
Designed molecule 2	12	Thr-26, Trp-27, Val-28, Asp-29, Leu-30, Val-31, Leu-32, Val-193, Val-195, Asp-196, Cys-197, Glu-198	−8.7	−7.67	54.55	−5.45	−2.00
Designed molecule 3	11	Thr-26, Trp-27, Val-28, Lys-45, Pro-46, Ile-48,Leu-187, Glu-192, Val-193, Thr-194, Asp-196	−7.7	−6.99	39.78	−5.34	−1.45
Designed molecule 4	14	Thr-26, Trp-27, Val-28, Glu-30, Ile-40, Asp-44 Lys-45, Pro-46, Ile-48, Leu-187, Tyr-190, Glu-192, Val-193, Thr-194	−8.9	−7.03	43.87	−7.89	−1.67

#### QSAR and pharmacoinformatics analysis

**Figure 12:**
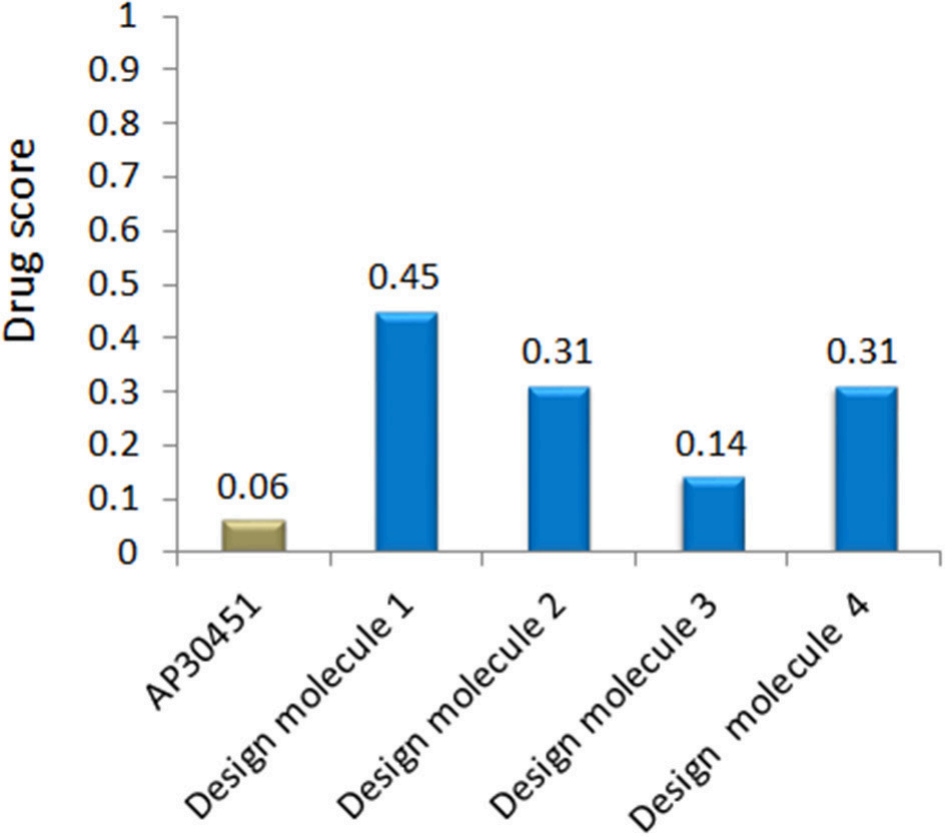
Drug score of WNV inhibitor.

The important criteria for establishing a new drug is to analyse the structural activity relationship and pharmacoinformatics analysis. We have exploited some bioinformatics tools (Described in Materials and Methods section) for the analysis of druggable properties which upsurge these designed inhibitors as potential drug candidates. We also analyzed ADME properties (Absorption, Distribution, Metabolism, Excretion) including Human Intestinal Absorption (HIA), Skin permeability, Blood-Brain Barrier distribution (LogBB), Caco-2 cell permeability, Volume of distribution, CYP450 2C9 substrate and inhibitor etc. The outcomes from these analyses meet the criteria for the new drug discovery. The overall toxicity (mutagenic, irritative, reproductive toxicity, and carcinogenic) of designed drugs were satisfactory than the mother compound AP30451. The drug-likeness and oral bioavailability confirmed that designed molecule 1 (drug score 0.45) could be the best choice for the potential drug among all the designed inhibitors along with AP30451 Figure 12 and Table 5.

**Table 5:** ADME properties of designed inhibitors.

**Properties**	**AP30451**	**Design molecule 1**	**Design molecule 2**	**Design molecule 3**	**Design molecule 4**
**ABSORPTION**					
Renal organic cation transporter	0.8723	0.9264	0.9358	0.9131	0.9054
P-glycoprotein Inhibitor	0.6948	0.9304	0.9374	0.9187	0.9430
Blood brain barrier	0.8642	0.5626	0.8377	0.5992	0.8757
Human intestinal absorption	0.9974	0.9740	0.9925	0.9862	0.9596
Caco-2 permeability	0.5178	0.6107	0.5714	0.5442	0.6098
**DISTRIBUTION**					
DBP (%PPB)	99.25%	96.1%	98.56%	99.11%	99.46%
Blood brain distribution(logBB)	−1.25	−0.81	−0.85	−1.25	−0.76
Volume of distribution(Vd)	2.17 L/kg	0.27 L/kg	0.35 L/kg	0.22 L/kg	0.2 L/kg
**METABOLISM**					
CYP450 2C9 substrate	0.6551	0.7126	0.6915	0.7898	0.6119
CYP450 2C9 inhibitor	0.6502	0.7270	0.7764	0.8533	0.5103
**TOXICITY**					
Mutagenecity	0.6	1.0	1.0	1.0	1.0
Tumorigenecity	0.6	1.0	1.0	1.0	1.0
Irritating effects	0.6	1.0	1.0	0.6	1.0
Reproductive effects	1	1.00	1.0	1.0	1.0

## Discussion

The underlying factors of recent emerging and re-emerging diseases include the microbial agent, human host and human environment (Morens and Fauci, 2013). Alarmingly, these sorts of diseases are now-a-days not confined to the developing countries. The infection of WNV is one of the emerging diseases and fatalities in humans and livestock around the globe. A few WNV vaccines in the pipeline provided a proof of concept for veterinary use. Still efforts on the development of effective therapeutics for human remain unsuccessful partly due to wet lab approaches which couldn't allow the faster method. However, the recent development of immunopharmacology tools together with the understanding of structure and function of WNV proteins could provide a faster and stronger platform for designing and developing vaccines/drugs as potential therapeutics. In the current study, we have been able to retrieve, store and utilize a lot of information about genomics and proteomics of WNV virus made available by the sequence-based technology which in turn allowed us the identification of epitopes from antigenic protein. The designing of epitope-based peptide vaccine against WNV achieved through the identification of a neutralizing epitope using similar bioinformatics tools applied for the recently recommended epitope-based vaccines such as dengue, chikungunya etc (Lapelosa et al., 2009; Chakraborty et al., 2010; Islam et al., 2012; Hasan et al., 2013; Hossain et al., 2018).

B-cell immunity is given the priority to design vaccine but T-cell was also shown to induce strong immune response (Van Joolingen et al., 2005). Also, T cell immune response is long-lasting immunity as foreign particles can avoid the effect of memory produced by an immune system. To suggest both B-cell and T-cell epitopes from the most antigenic protein we have used different bioinformatics tools to confer the immunity in WNV. A well-conserved T cell epitopes among the envelope glycoprotein sequences of WNV were considered as strong and potent. Our predicted human non-allergen T-cell epitope “KSFLVHREW” is expected to be effective as peptide vaccine as well as provide the protection broadly against various strains due to its higher binding affinity to interact highest numbers (13) of HLA and very high conservancy (82.46%) respectively Tables 1, 2, and Figure 6. This higher binding affinity of this epitope to MHC allele was confirmed by docking analysis by using a specific allele Figure 4. HLA alleles responsible for exposing affinity to KSFLVHREW were investigated for population coverage in WNV endemic regions. The significant level of population coverage was achieved for European, Asian and American population Figure 3. According to these results, it is obvious that this non-allergen vaccine would be effective for a vast population throughout a wide geographical region Figure 3. Through rigorous analysis of several characteristics such as the presence of antigenicity, beta-turns, flexibility, accessibility, linearity and hydrophilicity, this study also predicted, identified and confirmed a potent B-cell epitope “ITPSAPSYT” from WNV envelope glycoprotein Figures 5, 6. In addition to an effective vaccine, a universal drug is required for the minimization or complete elimination of chronic symptoms caused by the infection which may last for months to years. Further, sometimes the action of vaccine might not be effective due to acquired mutational change. Then the treatment with drugs could be the best choice against WNV. Nevertheless, vaccination won't be effective in the worst case of sudden WNV outbreak. Therefore, we have also identified the active sites of the highest antigenic protein of WNV for proposing some novel inhibitors alongside with pre-therapeutic vaccine design. Drug designing against WNV was based on the highest antigenic protein from its proteome, the envelope glycoprotein. Before designing the inhibitor, we have predicted the 3D model of the target glycoprotein based on a PSI blast identified model template 3P54_A and confirmed its superior quality Supplementary Figures S1–S4. We have designed four WNV inhibitors from a control WNV inhibitor AP30451 which upon structural stability analysis indicated their candidature as drugs Figures 7, 8. It is clearly apparent that all the designed molecules are the most potent inhibitors as they have lowest docking energy indicating their higher binding affinity to the envelope glycoprotein. Further, they interacted with active pocket amino acid Thr-26, Trp-27, and Val-193 of envelope glycoprotein Tables 3, 4 and Figures 9, 10. Besides, the mode of action of designed and control inhibitors was found similar when they were assessed for their pharmacological properties Table 5. Poor metabolism or toxic effect might urge to fail a drug compound in phase III clinical trials (Ju et al., 2011). The fundamental characteristics of the establishment of drugs are to measure their pharmacophore properties. The advancement of bioinformatics software could be employed to predict these properties before the wet lab confirmation (Ekins et al., 2007). Among the ADMET properties, all the designed molecules showed similar human intestinal absorption rate, higher metabolic rate, no sign to cross blood-brain barrier and balanced volume of drug distribution into the body. Furthermore, the toxic level of these compounds seems to be standard than the parent compound. Also the higher drug likeness of our designed compounds 1(0.45), 2(0.31), 3(0.14), and 4(0.41) over the experimentally validated control compounds AP30451 (0.05) support their effectiveness as inhibitory agents of the envelope glycoprotein of WNV Figure 12 and Table 5.

However, before the practical application of these predicted epitopes, experimental trials are necessary. For the inhibitors, the suggested compound has to be synthesized to test its efficacy in animal model experiments.

## Conclusion

Vaccination and drug administration protect human from serious illness and complications against the diseases. Therefore, we have employed bioinformatics approach to look for an effective method of preventing viral disease. The current study suggests that both T and B cell epitope might uphold the progressive stage of novel peptide vaccine discovery to possible treatment against WNV. In addition, inhibitors are designed against WNV envelope glycoprotein as post-therapy which could be suggested to experimental validation for the development of drugs. The *in silico* results confirmed that the predicted epitopes could elicit the immune response and designed drugs also could assist as therapeutic agents against WNV infection.

## Author contributions

MS: Conceived, designed, and guided the study, analyzed the data, drafting the manuscript and performed critical revision; CK: Guided the study, acquisition, and analyzed the data, helped in drafting the manuscript; KD and AH: Guided the study, analyzed the data, and helped in drafting the manuscript. TO, MK, and SR Helped in Bioinformatics analysis and helped in drafting the manuscript. MH: Helped to design the study, performed bioinformatics analysis, drafted, and developed the manuscript and performed critical revision. All authors have approved the manuscript.

### Conflict of interest statement

The authors declare that the research was conducted in the absence of any commercial or financial relationships that could be construed as a potential conflict of interest.

## Abbreviations

WNV: West Nile Virus
ALL: Acute Lymphoblastic Leukemia
RCSB: Research Collaboratory for Structural Bioinformatics
CADD: Computer Aided Drug Design
PDB: Protein data bank
3D: 3 dimensional
LogS: Logarithm of solubility
ADME/Tox, Absorption, Distribution, Metabolism, Excretion: Toxicity
TPSA: The polar surface area
cLogP: Logarithm of partition coefficient
HIA: Human Intestinal Absorption HIA
Vd: Volume of Distribution
QSAR: Quantitative structural activity relationship
UniPortKB: UniProt Knowledge Base
IC50: half-maximal Inhibitory Concentration
CTL: Cytotoxic T-lymphocyte
TAP: Transport Associated Proteins
IEDB: Immune Epitope Database
MHC: Major Histocompatibility Complex.
