# Supplementary figures and images for: An Immunopharmacoinformatics Approach in Development of Vaccine and Drug Candidates for West Nile Virus
Source: Front Chem. 2018 Jul 6;6:246. doi: 10.3389/fchem.2018.00246 (PMC6043868; doi:10.3389/fchem.2018.00246)

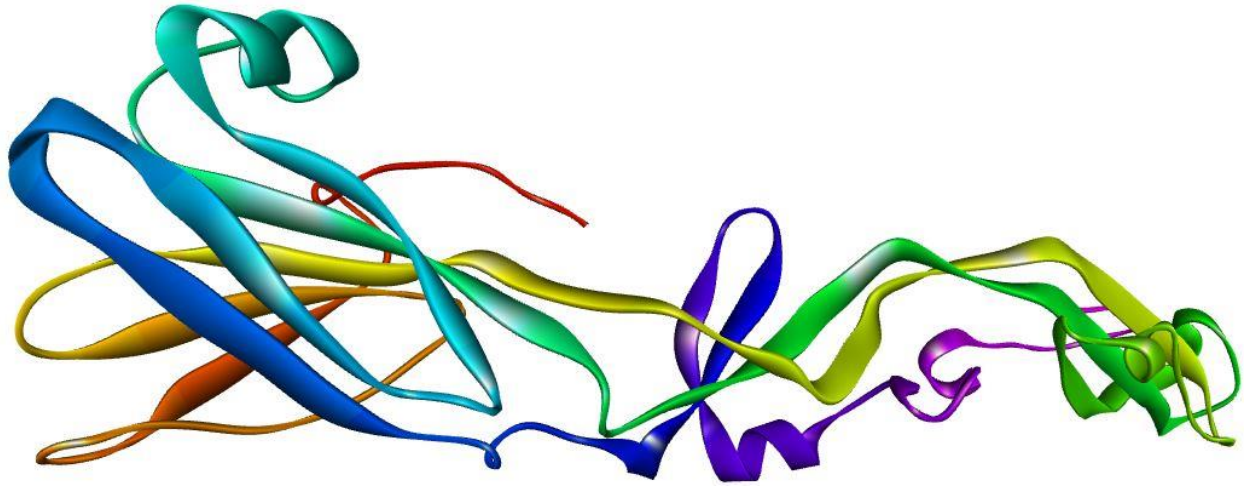

**Supplementary figure, S1: Predicted 3D structure of WNV envelope glycoprotein**

Supplement: Supplementary file 1 [file Image_1.PDF]

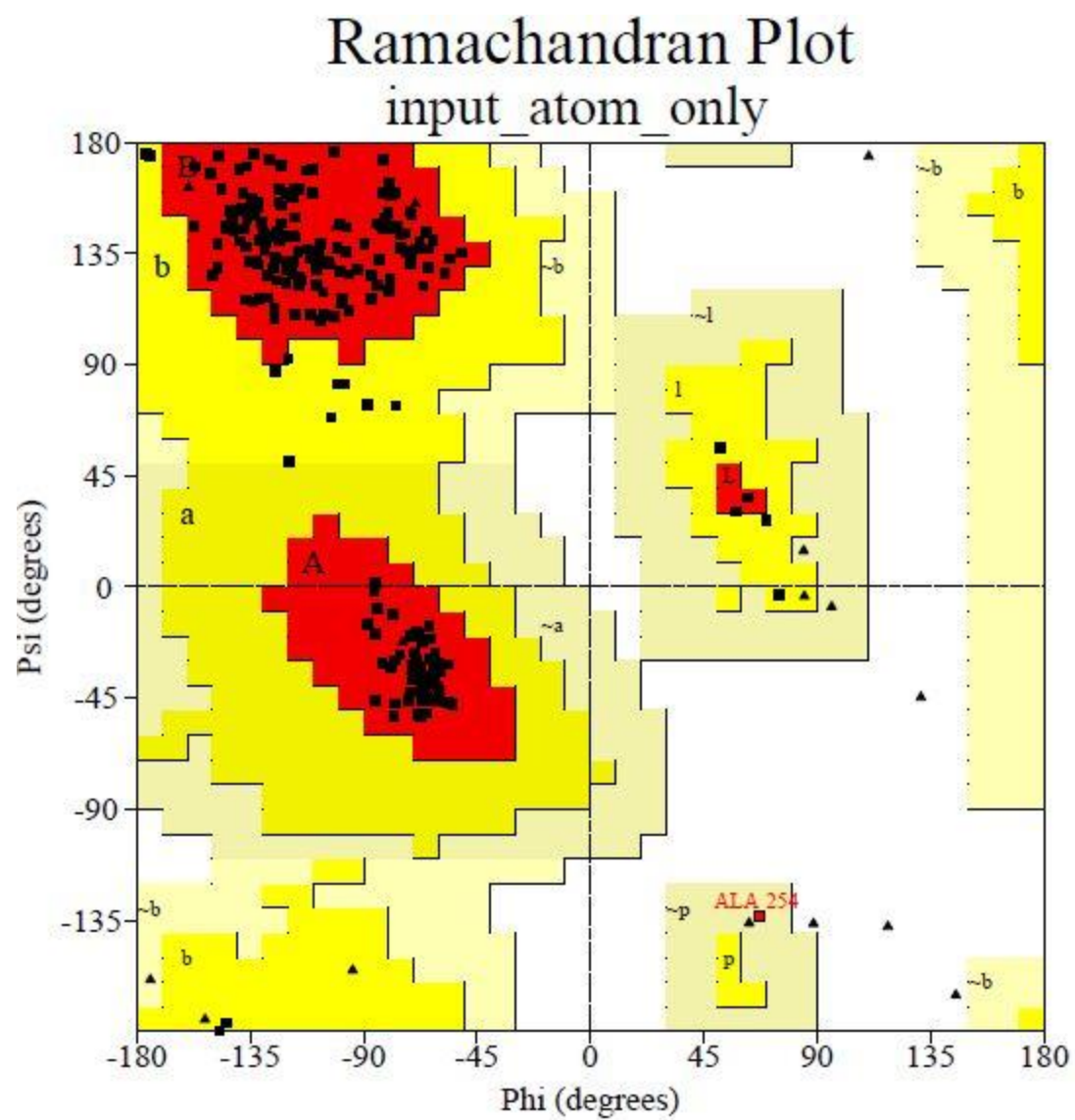

**Supplementary figure, S2:** Ramachandran plot for built model.

Supplement: Supplementary file 2 [file Image_2.PDF]

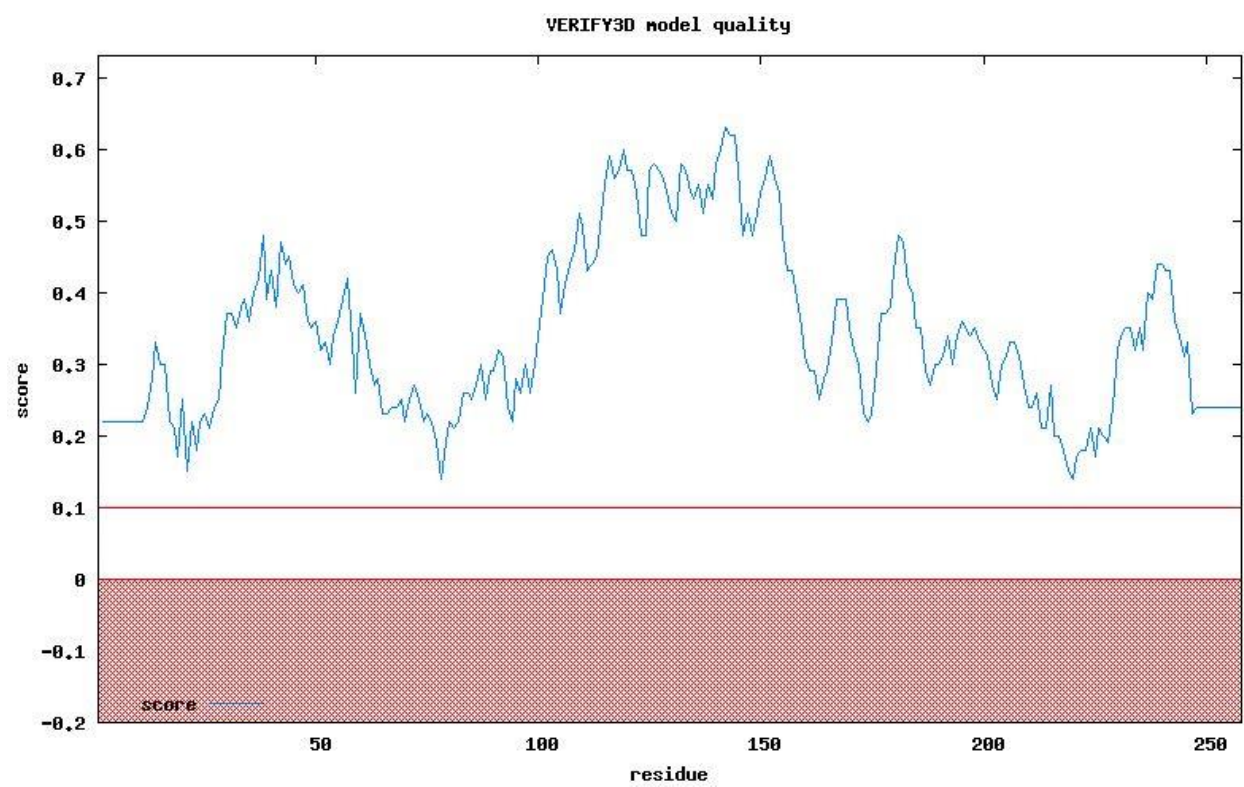

**Supplementary figure, S3:** VERIFY3D Model quality assessment for predicted model.

Supplement: Supplementary file 3 [file Image_3.PDF]

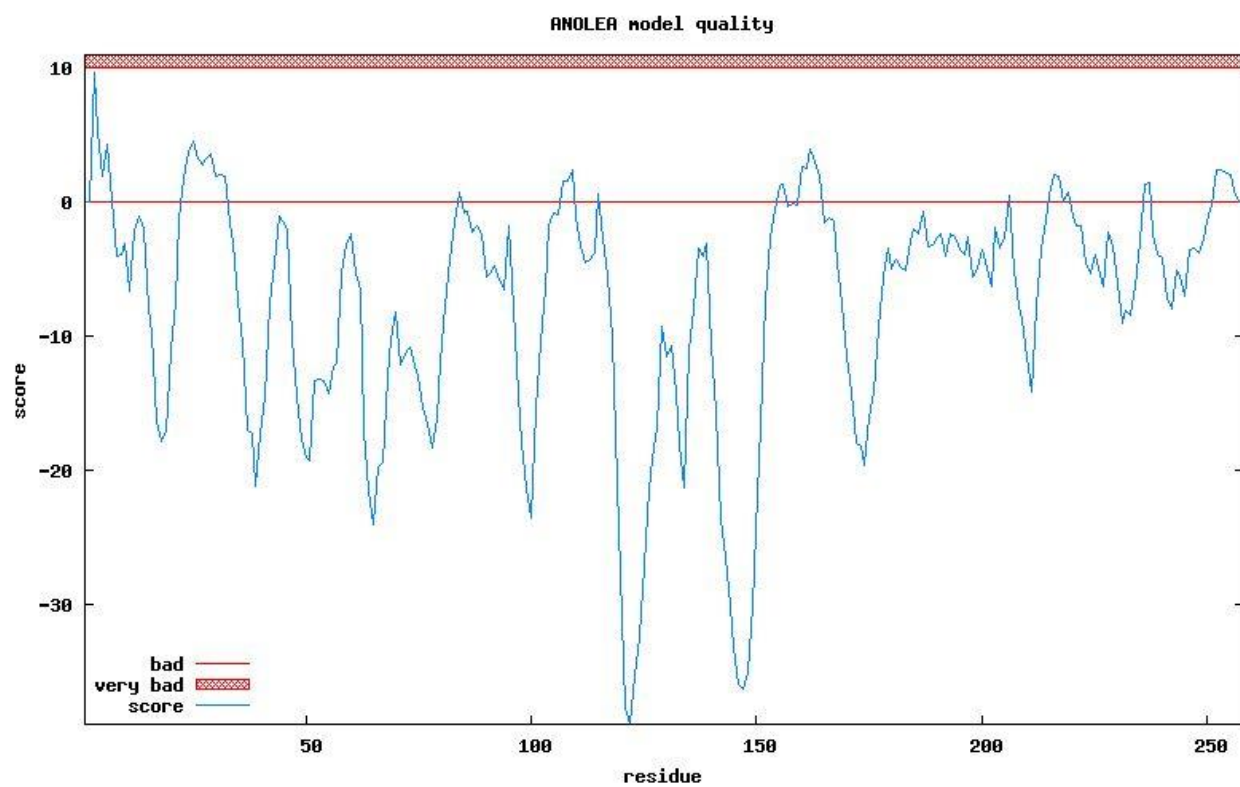

**Supplementary figure, S4:** ANOLEA Model quality assessment for predicted model.

Supplement: Supplementary file 4 [file Image_4.PDF]
