# Supplementary material for: An Immunopharmacoinformatics Approach in Development of Vaccine and Drug Candidates for West Nile Virus
Source: Front Chem. 2018 Jul 6;6:246. doi: 10.3389/fchem.2018.00246 (PMC6043868; doi:10.3389/fchem.2018.00246)
Supplement: Supplementary file 5 [file Table_1.PDF]

Supplementary Table, S1: Model quality assessment of Ramachandran plot.

**Ramachandran plot statistics**	**Residues**	**%**
Residues in the most favored regions [A,B,L]	208	92.9%
Residues in the additional allowed regions [a,b,l,p]	15	6.7%
Residues in the generously allowed regions [a,b,l,p]	1	0.4%
Residues in the disallowed regions [xx]	0	0.0%
Number of non-glycine and non-proline residues	224	100%
Number of end residues (excl. Gly and Pro)	2	
Number of glycine residues	22	
Number of proline residues	10	
Total number of residues	258	
